# Synthesis of Eco-Friendly Silver Nanoparticles Using Glycyrrhizin and Evaluation of Their Antibacterial Ability

**DOI:** 10.3390/nano12152636

**Published:** 2022-07-30

**Authors:** Danni Feng, Renyin Zhang, Mengting Zhang, Ashe Fang, Feng Shi

**Affiliations:** College of Life Science, Shihezi University, Shihezi 832003, China; fdanny@163.com (D.F.); zrenyin@163.com (R.Z.); zmtcue@163.com (M.Z.); fangashe@163.com (A.F.)

**Keywords:** silver nanoparticles, glycyrrhizin, antibacterial, green synthesis

## Abstract

In the present study, the biosynthesis of silver nanoparticles (AgNPs) and their antibacterial activity against gram-positive and gram-negative bacteria were investigated. Glycyrrhizin (GL) was used as a reducing agent and stabilizer to rapidly prepare the AgNPs. The distinctive absorption peak at 419 nm confirmed the formation of GL-reduced AgNPs. The TEM and particle size analysis shows that the prepared GL-reduced AgNPs were mostly circular with good dispersion and a relatively uniform particle size of 35 nm on average. Fourier transform infrared spectroscopy analysis was performed to identify the possible biomolecules in the capping and active stabilization of the GL-reduced AgNPs. The antibacterial activity of the GL-reduced AgNPs was analyzed with the Oxford cup diffusion method and filter paper diffusion method. The experimental results show that these properties endowed the GL-reduced AgNPs with high antibacterial activity against *Escherichia coli* and *Staphylococcus aureus* and lay a foundation for the use of colloidal silver in antibacterial applications. The GL-reduced AgNPs also had stronger antibacterial activity than sodium citrate-reduced AgNPs, which indicates the advantages of GL-reduced AgNPs compared with sodium citrate-reduced AgNPs in inducing bacteriostasis. The cytotoxicity of GL-reduced AgNPs on human kidney epithelial 293A (HEK293) cells was evaluated via the MTT assay. The results show that GL-reduced AgNPs had lower toxicity to HEK293 cells than sodium citrate-AgNPs, which indicates that the as-prepared GL-reduced AgNPs are environmentally friendly.

## 1. Introduction

Silver (Ag) is widely used by scientists who study biology and medicine [1]. Compared with other various nanoparticles (NPs), silver nanoparticles (AgNPs) have a high surface-to-volume ratio (size below 100 nm); therefore, modern nanomedicine can greatly benefit from the unique antimicrobial properties of nanoparticles [2,3]. However, conventional methods for AgNP synthesis require dangerous chemicals and high temperatures, which can result in the production of hazardous byproducts or the absorption of harmful chemicals on the surfaces of NPs and increase toxicity. There is a growing need for an ecologically friendly AgNP biosynthesis technology that does not produce toxic wastes during the synthesis process [4]. Hence, plant-based NP synthesis has attracted the interest of researchers due to its cost-effectiveness, ecological friendliness, and extensive applications. Glycyrrhiza is a widely used Chinese herbal medicine that has strong pharmacological properties, including anti-inflammatory, antiviral, and other effects. Glycyrrhizin (GL) is an important active substance in Glycyrrhiza with many pharmacological and biological activities, such as antitumor, anti-inflammatory, antioxidative, and antiviral activitis [5]. The use of plant extracts with antibacterial activity as reducing and capping agents is helpful for preparing NPs with enhanced antimicrobial activity.

Medical systems worldwide are facing serious obstacles due to the outbreak of resistant pathogens [6]. *Escherichia coli* (*E. coli*) is a type of gram-negative foodborne pathogen that can cause gastrointestinal tract infections or urethral infections in humans and animals under certain conditions [7]. *Staphylococcus aureus* (*S. aureus*), which is a common gram-positive pathogen, is considered the second most common cause of foodborne illness globally [8]. *S. aureus* is also a facultative anaerobe that causes superficial lesions on the surface of human skin, as well as various suppurative infections and toxinoses in humans [9]. Many types of antibiotics are available to combat gram-bacteria. However, the broad use of these antibiotics has led to bacterial resistance [10]. Therefore, the demand for new antimicrobials with good antibacterial effects and low costs is increasing. The antimicrobial nature of nanoparticles is the most exploited feature of nanoparticles in the medical field. Nanoparticles are primarily prepared from noble metals, with AgNPs being the most widely used. AgNPs are some of the most vital NPs in biomedical applications and have been reported to possess antiviral, anti-inflammatory, antibacterial, and antifungal properties [11].

Therefore, to synthesize AgNPs in a green manner, research has focused on synthesizing AgNPs using biomolecules with intrinsic antimicrobial action. Notably, AgNPs produced through mediation by glycyrrhizin require further evaluation of their antimicrobial properties to provide a green synthetic alternative. The present study aimed to develop a method to rapidly synthesize AgNPs using glycyrrhizin, a biological molecule with antimicrobial activity, and evaluate its cytotoxicity to human kidney epithelial 293A (HEK293) cells and antibacterial activity against *E. coli* and *S. aureus*. The relative cytotoxicity was determined by 3-(4,5-dimethylthiazol-2-yl)-2,5-diphenyltetrazolium bromide (MTT) assays. This method is an eco-friendly approach with potential applications.

## 2. Materials and Methods

### 2.1. Materials

Silver nitrate (AgNO_3_) was purchased from Sigma (St. Louis, MO, USA). Glycyrrhizin (molecular weight: 822.93) was obtained from Beijing Balinway Technology Co., Ltd. (purity ≥ 98%) Beijing, China. Sodium hydroxide (NaOH) and sodium citrate (molecular weight: 258.07) were purchased from Shanghai Macklin Biochemical Technology Co., Ltd. (purity ≥ 98%, Shanghai, China). *Escherichia coli* (*E. coli*) was provided by the Microbiology Laboratory, College of Life Sciences, Shihezi University, Shihezi, China. *Staphylococcus aureus* (*S. aureus*) was obtained from the BNCC and stored in our laboratory. Sterile LB medium was prepared in our laboratory. All other chemical reagents were of analytical grade and used as received. We used ultrapure water from a Milli-Q A10 water purification system for all experiments. The Oxford cup had an inside diameter of 6 mm and an outside diameter of 8 mm. Foetal bovine serum (FBS) was purchased from Shanghai Excell Biological Technology Co., Ltd. (Shanghai, China). The MTT assay kit was purchased from Beyotime Biotechnology Co., Ltd. (Shanghai, China). Dulbecco’s modified Eagle’s medium (DMEM), trypsin, and penicillin-streptomycin solution were purchased from HyClone Co., Ltd. (Logan, Utah, USA). The Thermo Forma Steri-Cycle i160 carbon dioxide incubator and human kidney epithelial 293A cells were purchased from Thermo Fisher Scientific Co., Ltd. (Waltham, MA, USA).

### 2.2. AgNP Synthesis

Silver nitrate (AgNO_3_) was used as a precursor, and GL was used as a reducing agent to synthesize AgNPs. A mixed solution of 5 mg of solid AgNO_3_ and 50 g of ultrapure water were placed in a conical flask in the beginning, and the reaction temperature and stirring speed were adjusted with a thermostatic magnetic stirrer. After boiling, 2 mL of GL solution (1%) was added to initiate the reaction. The pH of the mixture was adjusted with 1 M NaOH during the reaction. The mixture was continuously whisked at different temperatures and speeds until the solution turned yellow. The reaction was stopped after the color of the mixture remained unchanged for five minutes. The final stage of the reaction was marked with a visual color change and the appearance of surface plasmon resonance (SPR) bands in the UV-Vis spectrum. The experiments were repeated three times to verify the reproducibility of the AgNP preparation procedure.

Reaction temperature is a crucial factor that affects the nucleation and size of NPs [12]. The stirring speed was also found to affect the nucleation and aggregation of AgNPs. Li and Kaner (2006) showed that stirring triggered heterogeneous nucleation and induced aggregation [13]. Therefore, the effects of various hotplate temperatures (100 °C, 150 °C, 200 °C, 250 °C, the stirring speed was set to 400 rpm) and stirring speeds (280 rpm, 400 rpm, 530 rpm, 750 rpm, the temperature of the hotplate is set to 200 °C) on the synthesis of AgNPs were studied. The measurement of solution temperature in a container is prone to large deviation; hence, the temperature stated here is the temperature of the hotplate. Pure water was added to supplement the evaporated solvent at the end of the reaction.

Sodium citrate-colloidal silver was prepared according to P. C. Lee’s method with a slight modification to compare its antibacterial ability with that of glycyrrhizin-colloidal silver [14].

### 2.3. AgNP Characterization

Parameters such as the morphology, particle size and dispersion of the AgNPs, as well as other properties, were characterized with an enzyme-labelled instrument, a Malvern particle size analyzer, FT-IR and TEM.

The as-prepared colloidal silver (3 mL) was analyzed in quartz cuvettes with an enzyme-labelling instrument using distilled water as a reference solvent. The absorption of each sample was measured at wavelengths of 300–700 nm and a resolution of 1 nm to measure the SPR and obtain the absorbance spectra. The maximum absorption peak wavelength and peak width were determined.

The particle size distribution and zeta potential of the AgNPs were measured with a Malvern particle size analyzer to characterize the mean particle size, particle size distribution and stability of the colloidal dispersion system.

The samples were prepared by drop coating a colloidal solution of silver nanoparticles (5 mL) onto a hydrophilic carbon-coated copper grid. After drying at room temperature, the mean particle size, surface morphology and dispersion of the as-prepared silver nanoparticles were determined by TEM.

The prepared colloidal silver was centrifuged at 10,000 rpm for 30 min, and the precipitate was ground to a fine powder, which was evenly mixed with dried KBr and pressed into thin pellets. Then, FT-IR analysis was conducted over a scanning range of 400–4000 cm^−^^1^.

### 2.4. Antibacterial Activity Assay

In the present investigation, the antimicrobial potential of AgNPs prepared with GL as a reducing agent against *E. coli* and *S. aureus* was investigated. The antimicrobial activity was evaluated using the Oxford cup method.

To prepare the inoculum in a sterile environment, isolated colonies of the two types of bacteria were selected and incubated in Luria-Bertani (LB) liquid medium for 24 h at 37 °C. The bacterial suspension was diluted to 10^8^ cfu/mL, and 0.2 mL of each diluted bacterial culture suspension was inoculated on LB plates and evenly spread with a sterile coater.

The Oxford cup diffusion method was performed by creating four vertical holes in the Oxford cup on each plate equidistant apart, and 0.1 mL of each sample was added to each hole. The prepared glycyrrhizin-colloidal silver was added to the left hole, GL solution at the same concentration was added to the top hole, sodium citrate-colloidal silver at the same concentration was added to the right hole, and distilled water was added to the bottom hole as a control. The samples on the plate were allowed to diffuse for half an hour. Then, the plate was incubated at 37 °C for 24 h, and the diameter of the circular antibacterial zone was measured to determine the bacteriostatic function of the AgNPs.

The experimental steps of the filter paper diffusion assay were as follows. First, several circles with diameters of 6 mm were punched into the filters. After autoclave sterilization, the filter papers were immersed in sample solution for half an hour. In a sterile environment, sterilized tweezers were used to pick up the filter papers, which were placed equidistant on plates coated with bacterial culture suspension. Similar to the Oxford cup diffusion method, each plate contained four circular filter papers immersed in glycyrrhizin-colloidal silver, sodium citrate-colloidal silver, GL solution and distilled water.

All antibacterial activity tests were performed in triplicate to certify their reproducibility.

### 2.5. Assessment of Cytotoxicity by MTT Assay

We studied the cytotoxic properties of glycyrrhizin-reduced AgNPs and sodium citrate-reduced AgNPs at different concentrations. Human embryonic kidney 293A (HEK293) cells obtained from Invitrogen were used in this experiment [15]. An MTT colorimetric assay was used to evaluate the cytotoxic effect of AgNPs on HEK293 cells with a 96-well plate [16]. HEK293 cells were cultured in DMEM with 10% FBS and 1% penicillin-streptomycin meidum at 37 °C in a humidified atmosphere with 5% CO_2_ for 2–3 days. Culture passage was conducted when the cell density reached 70–80%. HEK293 cells in logarithmic growth phase were trypsinized, and the cell density was adjusted to 10^5^ cells/mL with complete culture medium. The cell suspensions were seeded in 96-well culture plates and cultured for 24 h. Glycyrrhizin-reduced AgNPs and sodium citrate-reduced AgNPs of different concentrations were sterilized through a 0.22-μm filter and mixed with the medium to a ratio of 1:1, and 100 μL of the mixture was placed into a 96-well plate so that the final mass concentration of the AgNPs was 20, 40, 60, 80, 100 μg/mL. The mixture was cultured in an incubator at 37 °C in a humidified atmosphere with 5% CO_2_ for 24 h, and the cell activity was detected according to the instructions for the MTT kit.

## 3. Results

### 3.1. Synthesis and Characterization of the Glycyrrhizin-Reduced AgNPs

**Figure 1:**
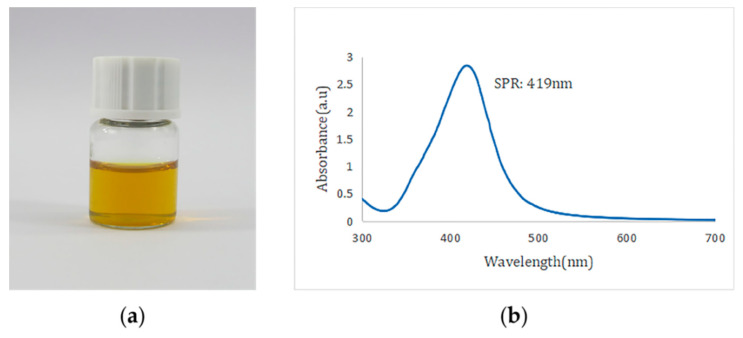
(**a**) Glycyrrhizin-reduced AgNPs in natural light, (**b**) UV-Vis spectra of glycyrrhizin-reduced AgNPs.

**Figure 2:**
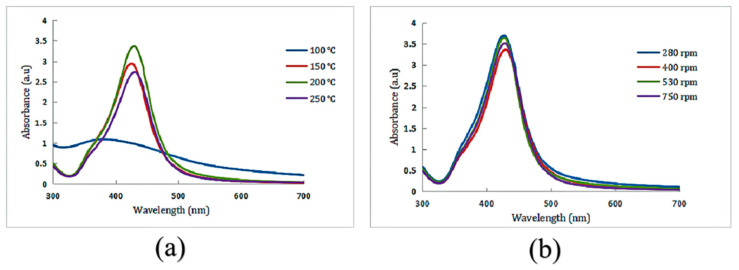
(**a**) UV-Vis spectra of glycyrrhizin-reduced AgNPs at different hotplate temperatures, (**b**) UV-Vis spectra of glycyrrhizin-reduced AgNPs at different stirring speeds.

Visually, the color change from colorless to yellow confirmed the formation of AgNPs (Figure 1a), which was further supported by the SPR band. The colloidal silver prepared under several different reaction conditions appeared as different shades of yellow under natural light. However, they were all clear and transparent without precipitation. SPR bands can provide useful information about the size and shape of the synthesized nanoparticles. The formation of AgNPs in the reaction mixture was verified by UV-Vis spectroscopy, which showed an SPR band at wavelengths between 300 and 700 nm. The effects of various hotplate temperatures (100 °C, 150 °C, 200 °C, 250 °C) and stirring speeds (280 rpm, 400 rpm, 530 rpm, 750 rpm) on the synthesis of AgNPs were studied (Figure 2). As shown in Figure 2a, there is only a slight fluctuation in the curve at 100 °C. The half-peak widths of the UV-Vis spectra at the other three temperatures are basically equal, with peak values at 424–430 nm. The higher the temperature is, the larger the corresponding peak. As shown in Figure 2b, the half-peak widths of the UV-Vis spectra under the four stirring speeds are basically identical, and the peak values are 424–430 nm, which indicates that the stirring speed hardly affects the peak value and the liquid concentration. The optimal preparation conditions of glycyrrhizin-colloidal silver were finally determined as follows: in a 50-mL conical flask, 5 mg AgNO_3_ was poured into 50 g ultrapure water and mixed well. The speed of the magnetic stirrer was set at 530 rpm, and the temperature was adjusted to 200 °C. Then, 2 mL of 1% glycyrrhizin solution was added for the reduction reaction to obtain glycyrrhizin-colloidal silver, and the pH of the mixture was adjusted to approximately 6.5. The characterization results of the as-prepared glycyrrhizin-colloidal silver are as follows:

The UV-Vis spectrum indicated an SPR peak at 419 nm (Figure 2b), which confirmed the production of AgNPs [17,18]. The AgNPs remained stable without aggregation and precipitation after being placed in a 4 °C refrigerator for one month, there was no significant change in the position of the absorption peak or the maximum peak in the UV-Vis spectrum, which indicates stability of the formed AgNPs (The SPR after one month of placement: 425 nm).

**Figure 3:**
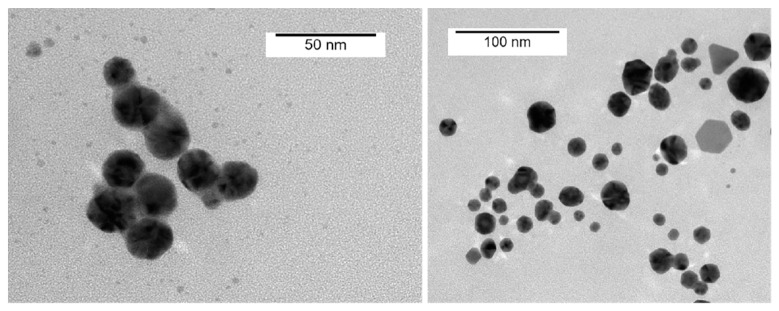
TEM images of the GL-reduced AgNPs at different scales.

To characterize the shape, dimensions, and size distribution of the AgNPs, transmission electron microscopy (TEM) measurements were performed. The TEM images of the AgNPs synthesized in this study showed spherical particles with an average size of 35 nm, while a few particle aggregates were also observed (Figure 3). The shape of the nanoparticles analysed using TEM was almost spherical or oval, and a few triangular and polygonal-shaped AgNPs were also found. Thus, the TEM images showed that the AgNPs were approximately spherical with a relatively uniform particle size, good dispersion, and low agglomeration, which indicates moderate stability of the prepared AgNPs due to the capping stabilization effect of the GL molecules.

**Figure 4:**
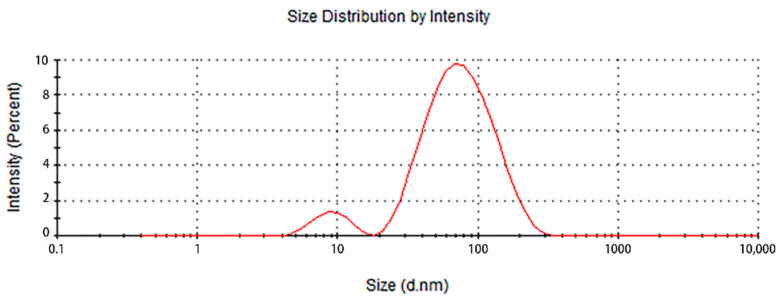
Particle size distribution of the GL-reduced AgNPs.

The particle size analysis results of the prepared colloidal silver samples are shown in Figure 4. Two peaks appeared in the particle size analysis diagram, which indicates a fraction of AgNPs with a particle size of 7–10 nm, while a larger fraction of the AgNPs was between 50–90 nm, for an average particle size of 52 nm. The polydispersity index (PDI) was 0.432, which indicates that the size distribution of the AgNPs in the colloidal silver was relatively concentrated. Then, the zeta potential was measured to obtain information about the surface charge and stability of the as-prepared AgNPs.

**Figure 5:**
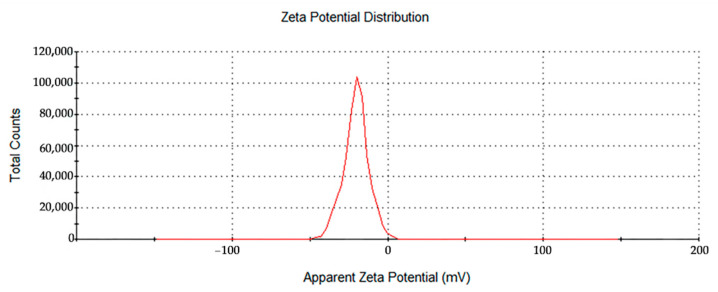
Zeta potential distribution of the GL-reduced AgNPs.

The zeta potential is an important parameter to characterize the stability of colloidal dispersions. The zeta potential of a sample determines whether the particles are stable or tend to flocculate and stick together when suspended in solution. If the absolute value of the zeta potential is large, the nanoparticles repel one another, which provides stability to the entire system. In this study, the synthesized AgNPs showed moderate stability. The zeta potential of the synthesized AgNPs was between −16.6 and −26.3 mV (Figure 5), which corresponds to a relatively stable colloidal suspension with a low tendency to form aggregatesF. The negative zeta potential value indicates the presence of GL molecules on the surface of the AgNPs.

**Figure 6:**
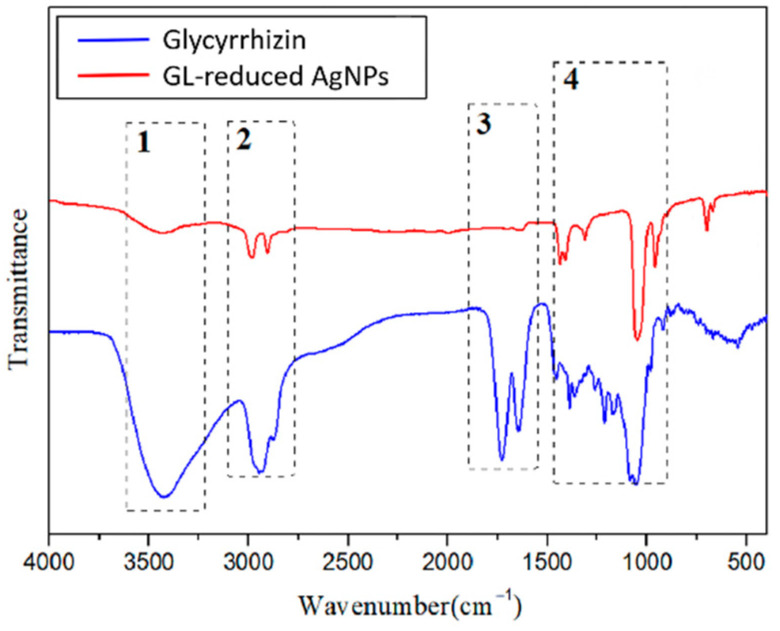
FT-IR spectra of glycyrrhizin-reduced AgNPs and glycyrrhizin.

Fourier transform infrared (FT-IR) spectroscopy was used to investigate GL and the synthesized AgNPs with GL at a resolution of 4 cm^−1^, and possible roles of the functional groups were analysed, as shown in Figure 6. The FT-IR spectrum of GL included typical bands at 3427, 2933, 1730, and 1647 cm^−1^ (dotted line square 1, 2, 3 and 4). The broad peak at 3427 cm^−1^ indicated the hydroxy group stretching vibration, which could be attributed to the presence of alcoholic hydroxyl groups in GL that might be involved in the reduction process. The signal at 2933 cm^−1^ indicated a carboxyl group. Additional intense peaks were observed at 1730 cm^−1^, which generally correspond to C=O stretching associated with a carbonyl group. After the bioreduction of AgNO_3_ by GL, notable shifts were observed in the FT-IR spectrum of the AgNPs. Major differences between the two spectra were observed at 3427 cm^−1^ and 2933 cm^−1^. These two peaks indicate the role of O-H bonds and C=O bonds in the synthesis of AgNPs. The peaks at 3433, 2981 and 2906 cm^−1^ in Figure 6 show that after the synthesis of the AgNPs, the stretching vibration signals from the hydroxyl and carboxyl groups were greatly weakened. The small fluctuation at 1643 cm^−1^ shows that the signal from the carbonyl stretching vibration almost disappeared. These data indicate that these functional groups were involved in the reduction process. Thus, the FT-IR spectral analysis clearly reveals that the alcoholic hydroxyl and carboxyl groups were the main functional groups in the GL-mediated synthesis of AgNPs.

### 3.2. Characterization of the Sodium Citrate-Reduced AgNPs

**Figure 7:**
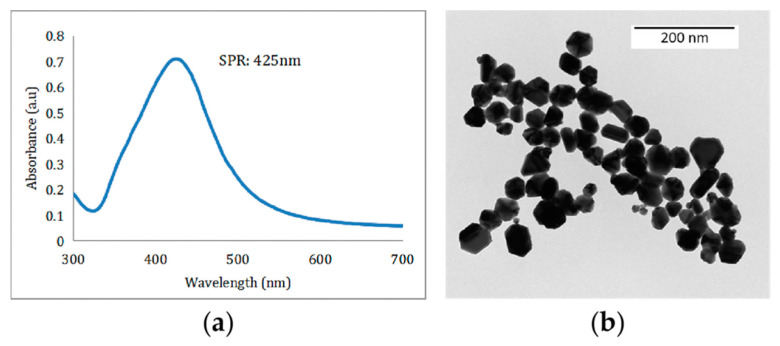
(**a**) UV-Vis spectra of sodium citrate-reduced AgNPs, (**b**) TEM image of the sodium citrate-reduced AgNPs.

The prepared Sodium Citrate-reduced AgNPs were tawny under natural light. It is not transparent enough but there was no precipitation. UV-Vis spectra analysis showed that there was an SPR peak at 425 nm (Figure 7a). Transmission electron microscopy analysis results (Figure 7b) showed that the particle size of the prepared Sodium Citrate-reduced AgNPs is relatively uniform about 60 nm, and the morphology is basically oval, with less agglomeration and good dispersion.

### 3.3. Antibacterial Activity Assay

The antibacterial activities of the AgNPs against *E. coli* and *S. aureus* were evaluated with the Oxford cup diffusion method and filter paper diffusion method, respectively. The antibacterial activity was reflected by the bacterial inhibition diameter (mm), where a larger antibacterial diameter corresponds to a better antibacterial effect. The results are shown in Table 1 and Table 2. As shown in the tables, almost all of the glycyrrhizin-reduced AgNPs produced a large or small clear inhibition zone. There was an inhibition zone around it only when the concentration of sodium citrate-reduced AgNPs was high, while the distilled water spot and glycyrrhizin solution spot were surrounded by bacteria. These data show that GL could inhibit bacteria far less than colloidal silver prepared by using GL as a reducing agent, and GL-reduced AgNPs had stronger antibacterial activity than sodium citrate-reduced AgNPs, which indicates the advantages of GL-reduced AgNPs compared to sodium citrate-reduced AgNPs in bacteriostasis. Moreover, with increasing GL-reduced AgNP concentrations (20, 60, 100 μg/mL), the inhibition diameters of the two types of bacteria treated with GL-reduced AgNPs increased, which indicates that the antibacterial activity of the GL-reduced AgNPs was positively correlated with their concentration.

According to the tables, the *E. coli* inhibition diameter produced by 100 μg/mL GL-reduced AgNPs was 22 and 16 mm using the Oxford cup diffusion method and filter paper diffusion method, respectively. Similarly, the *S. aureus* inhibition diameter after treatment with 100 μg/mL GL-reduced AgNPs by the Oxford cup diffusion method and filter paper diffusion method was 18 and 14 mm, respectively, which indicates that the inhibitory effects measured by the Oxford cup diffusion method were superior to those measured by the filter paper diffusion method under identical conditions.

Through the use of the filter paper diffusion method, the inhibition diameters created by 20 μg/mL GL-reduced AgNPs on the two types of bacteria were very small, which indicates that, at lower concentrations, the GL-reduced AgNPs had almost no inhibitory effect against these bacteria. In the tables, the inhibition diameter of 100 μg/mL GL-reduced AgNPs with *S. aureus* was 18 mm, but the inhibition diameter with *E. coli* reached as high as 22 mm with the Oxford cup diffusion method. Using the filter paper diffusion method, the inhibition diameter of 100 μg/mL GL-reduced AgNPs with *S. aureus* was 16 mm, but the inhibition diameter with *E. coli* reached 14 mm. Other concentrations of GL-reduced AgNPs showed inhibition diameters that followed the same pattern for these two bacteria.

According to the tables, the sodium citrate-reduced AgNPs had slightly smaller growth inhibition diameters than the glycyrrhizin-reduced AgNPs at both low and high concentrations. The *E. coli* inhibition diameter produced by 100 μg/mL GL-reduced AgNPs was 22 mm by the Oxford cup diffusion method, while this value of Sodium Citrate-reduced AgNPs was only 12 mm. The *S. aureus* inhibition diameter produced by 100 μg/mL GL-reduced AgNPs was 18 and 14 mm using the Oxford cup diffusion method, and sodium citrate-reduced AgNPs, respectively. All of the above results show that GL-reduced AgNPs had stronger antibacterial activity than sodium citrate-reduced AgNPs, which indicates the advantages of GL-reduced AgNPs compared to sodium citrate-reduced AgNPs in bacteriostasis.

### 3.4. Assessment of Cytotoxicity by MTT Assay

**Figure 8:**
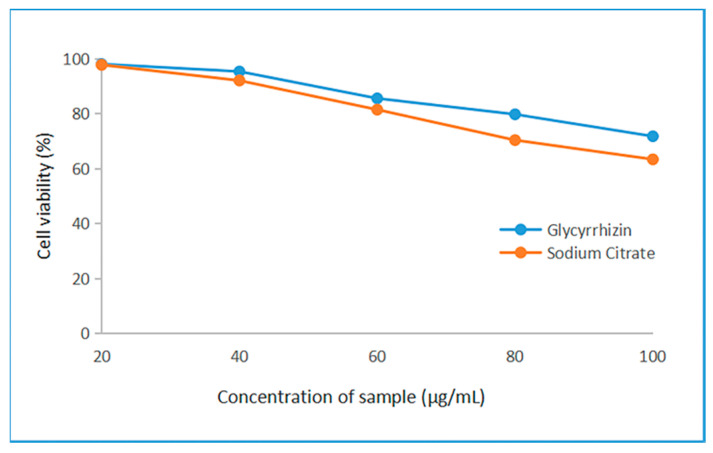
Cell viability of HEK293 cells in different concentrations of glycyrrhizin-reduced AgNPs and sodium citrate-reduced AgNPs.

An MTT colorimetric assay was used to evaluate the cytotoxic effect of glycyrrhizin-reduced AgNPs and sodium citrate-reduced AgNPs on HEK293 cells. The cytotoxicity of glycyrrhizin-reduced AgNPs and sodium citrate-reduced AgNPs to HEK293 cells at different concentrations is shown in Figure 8. The glycyrrhizin-reduced AgNPs had lower cytotoxicity to HEK293 cells than sodium citrate-reduced AgNPs. The glycyrrhizin-silver nanoparticles had higher cellular activity than sodium citrate-silver nanoparticles. At a low concentration of 20 μg/mL, neither AgNP showed cytotoxicity to HEK293 cells. At moderate concentrations of 40 μg/mL and 60 μg/mL, the cytotoxicity of AgNPs to HEK293 cells began to appear, but the cytotoxicity of glycyrrhizin-reduced AgNPs on 293 cells was lower than that of sodium citrate-reduced AgNPs. The cytotoxicity of glycyrrhizin-reduced AgNPs to HEK293 cells was much lower than that of sodium citrate-reduced AgNPs at high concentrations of 80 μg/mL and 100 μg/mL.

The results show that AgNPs at low levels had no cytotoxicity to HEK293 cells. When the concentration of the AgNPs increased, the cytotoxicity to HEK293 cells began to appear. The glycyrrhizin-reduced AgNPs had lower cytotoxicity to HEK293 cells than sodium citrate-reduced AgNPs, which indicates that the AgNPs prepared by this method are better than the AgNPs prepared by the traditional method, which is an eco-friendly synthesis method.

## 4. Discussion

In recent years, the green synthesis of AgNPs using different plant components has received considerable attention because of its simplicity, low cost, safety, and eco-friendly nature [13]. Sabah Ansard et al. prepared eco-friendly AgNPs with an average diameter of 20 nm from *Brassica oleracea* (BO). These BO-AgNPs had the highest antibacterial activity against *Staphylococcus epidermidis* (gram-positive) and *Pseudomonas aeruginosa* (gram-negative) [19]. Anush Aghajanyan et al. synthesized 20~90 nm AgNPs using an *Artemisia annua L.* extract as a reducing and stabilizing agent and demonstrated that a lower concentration of biogenic AgNPs exhibited antibacterial activity against gram-negative *Escherichia coli* BW 25,113 and gram-positive *Enterococcus hirae* ATCC 9790 [20]. The plants *Allium cepa* and *Allium sativa* (garlic) were used to prepare AgNPs with an average size of 42.14 nm by Nahla Alsayed Bouqellah et al., and the antibacterial actions of these AgNPs against the vaginal pathogens *Streptococcus pneumoniae* and *Pseudomonas aeruginosa* were confirmed [21]. In this study, glycyrrhizin, which is the active substance in Glycyrrhiza, was used as a reducing agent and stabilizer to prepare spherical AgNPs with good dispersion and uniform particle size that was 35 nm on average. The UV-Vis spectrum showed an SPR peak from the AgNPs at 419 nm. The zeta potential of the synthesized AgNPs indicated a relatively stable colloidal suspension. The FT-IR spectral analysis revealed that alcoholic hydroxyl and carboxyl groups were the main functional groups in the GL-mediated synthesis of AgNPs.

The prepared AgNPs in this study also showed good antibacterial performance. Previous studies have reported that AgNO_3_ shows stronger antibacterial properties than AgNPs [22]. However, silver in its ionic form is more toxic [23]. Considering the nontoxicity and biocompatibility of the prepared materials, biogenic AgNPs were determined to be the preferred material for subsequent antibacterial applications in this study. Although the exact mechanism of antibacterial action is not well understood, several factors influence the antibacterial action of nanoparticles. The particle size is an important factor that affects the antibacterial activity. In general, smaller nanoparticles have a larger surface area to volume ratio, provide a higher interaction area and have relatively high stability and strong antibacterial activity [3,24]. The size of nanoparticles should be lower than 50 nm to have effective antibacterial activity [25]. The average particle size of the GL-reduced AgNPs in this study was 35 nm, and the bacteriostatic test shows that they have good bacteriostatic activity, which is consistent with the previous literature. The zeta potential also affects the antimicrobial activity. The zeta potentials of the GL-reduced AgNPs in this paper ranged from −16.6 to −26.3 mV. The interaction between AgNPs and microorganisms is based on the electrostatic attraction between the negatively charged microbial cell walls or membranes and positively charged Ag^+^ released by Glycyrrhizin-reduced colloidal silver [26,27].

Kim et al. reported that the antibacterial activity of AgNPs was more effective against *E. coli* than against *S. aureus* [28]. Our results confirmed these findings, as GL-reduced AgNPs had higher antibacterial activity against *E. coli* than against *S. aureus*. This result may be attributed to different cell wall compositions of gram-positive and gram-negative bacteria. The cell walls of gram-positive bacteria have a rigid network of thick peptidoglycan layers that prevents nanoparticle penetration, while gram-negative bacteria are thin and consist of lipopolysaccharides [6,29]. Therefore, most AgNPs exhibit higher antibacterial activity against gram-negative bacteria than against gram-positive bacteria [30].

## 5. Conclusions

In this study, we report an eco-friendly method to synthesize of AgNPs based on the reduction of AgNO_3_ by glycyrrhizin. The antiseptic activity of the nanoparticles against certain gram-positive and gram-negative bacteria, such as *S. aureus* and *E. coli*, was studied. The cytotoxicity assays show that the cytotoxicity of glycyrrhizin-reduced AgNPs to HEK293 cells was lower than that of sodium citrate-reduced AgNPs, which indicates that the as-prepared GL-reduced AgNPs are environmentally friendly. The current synthetic method is simple, efficient, economical and environmentally benign. It also avoids the use of toxic chemicals, so the applicability of these AgNPs can be extended to various fields, such as those involving the environment and biomedicine. GL-reduced AgNPs displayed high antibacterial activity against *E. coli* and *S. aureus*, which indicates the advantages of green chemistry on the antimicrobial activity of silver nanoparticles. This study laid a foundation for future applications of silver nanoparticles in bacteriostasis. Despite the high antibacterial activity of the GL-reduced AgNPs, we suggest that further studies are required before other biological applications of AgNPs can be investigated.

## Figures and Tables

**Table 1:** Bacteriostatic diagram of GL-reduced AgNPs (*E. coli*).

*E. coli*	Glycyrrhizin-Reduced AgNPs Concentrations		
	20 μg/mL	60 μg/mL	100 μg/mL
Oxford cup diffusion method	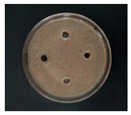	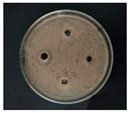	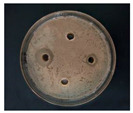
Growth inhibition diameter	10 mm	18 mm	22 mm
Filter paper diffusion method	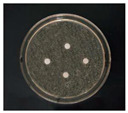	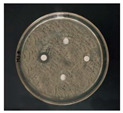	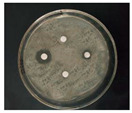
Growth inhibition diameter	6 mm	12 mm	16 mm

^1^ The outer diameter of the Oxford cup is 8 mm, and the diameter of filter paper is 6 mm. The liquid in the plate was GL-reduced colloidal silver (left), GL solution (top), sodium citrate-reduced AgNPs (right) and distilled water (bottom).

**Table 2:** Diagram of the bacteriostatic effect of GL-reduced AgNPs (*S. aureus*).

*S. aureus*	Glycyrrhizin-Reduced AgNPs Concentrations		
	20 μg/mL	60 μg/mL	100 μg/mL
Oxford cup diffusion method	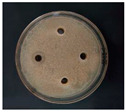	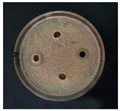	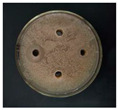
Growth inhibition diameter	12 mm	16 mm	18 mm
Filter paper diffusion method	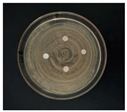	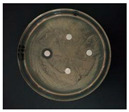	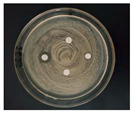
Growth inhibition diameter	6 mm	12 mm	14 mm

^1^ The outer diameter of the Oxford cup is 8 mm, and the diameter of filter paper is 6 mm. The liquid in the plate was: GL-reduced colloidal silver (left), GL solution (top), sodium citrate-reduced AgNPs (right) and distilled water (bottom).

## Data Availability

Not applicable.
